# The efficacy and safety of acupoint application combined with western medicine for allergic rhinitis: A protocol for systematic review and meta-analysis

**DOI:** 10.1097/MD.0000000000021627

**Published:** 2020-08-07

**Authors:** Yao Huang, Yihua Fan, Chunying Tian, Mengni Zhang, Shasha Yang, Yue Ji, Qinxiu Zhang

**Affiliations:** a Hospital of Chengdu University of Traditional Chinese Medicine; b Chengdu University of Traditional Chinese Medicine, Chengdu, Sichuan province; c Tianjin University of Traditional Chinese Medicine, Tianjin; d First Affiliated Hospital of Guizhou University of Traditional Chinese Medicine, Guiyang, Guizhou province; e School of Medical and Life Sciences/Reproductive & Women-Children Hospital, Chengdu University of Traditional Chinese Medicine, Chengdu, China.

**Keywords:** allergic rhinitis, acupoint application, efficacy, safety, systematic review

## Abstract

**Background:**

Acupoint application combined with western medicine has been used for treating allergic rhinitis widely. However, the efficacy and safety of acupoint application combined with western medicine for allergic rhinitis are unclear. This study aims to evaluate the efficacy and safety of acupoint application combined with western medicine for allergic rhinitis.

**Methods:**

Randomized controlled trials of acupoint application combined with western medicine for allergic rhinitis will be searched in PubMed, EMbase, Cochrane Library, Web of Science, China National Knowledge Infrastructure, WanFang, the Chongqing VIP Chinese Science and Technology Periodical Database, and China biomedical literature database from inception to July, 2020. And Baidu Scholar, Google Scholar, International Clinical Trials Registry Platform, and Chinese Clinical Trials Registry will be searched to obtain more relevant studies comprehensively. Two researchers will perform data extraction and risk of bias assessment independently. Statistical analysis will be conducted in RevMan 5.3.

**Results:**

This study will summarize the present evidence by exploring the efficacy and safety of acupoint application combined with western medicine for the treatment of allergic rhinitis.

**Conclusions:**

The findings of the study will provide helpful evidence for the efficacy and safety of acupoint application combined with western medicine in the treatment of allergic rhinitis, facilitating clinical practice and further scientific studies.

**Ethics and dissemination:**

The private information from individuals will not publish. This systematic review also will not involve endangering participant rights. Ethical approval is not required. The results may be published in a peer-reviewed journal or disseminated in relevant conferences.

**OSF Registration number:**

DOI 10.17605/OSF.IO/NSGJH

## 1 Introduction

Allergic rhinitis (AR) is a chronic inflammatory disease of nasal mucosa from an immunoglobulin E (IgE)-mediated immunological reaction to allergen exposure.^[1]^ The clinical symptoms of AR are rhinorrhea, nasal pruritus, nasal congestion and excessive sneezing, caused by common allergens including house dust mites, animal dander and pollen from grasses, trees and weeds.^[2,3]^ Epidemiological studies have found that AR affects up to 40% of the world's population and 11.1% to 17.6% of China's population, affecting patients’ quality of life and causing serious economic burden.^[4–8]^ The pathogenesis of AR is the imbalance of Th1 and Th2 cell-mediated inflammation.^[9,10]^ Due to the lack of complete cure, symptom control has traditionally been the main goal of AR treatment.^[11]^ At present, the mainstream of AR treatment includes avoiding allergens, western medicine therapy (glucocorticoid, antihistamines, et al), immunotherapy and desensitization.^[12]^ Although western medicine therapy can relieve symptoms rapidly, it becomes less effective due to the drug resistance. These treatments are related to the occurrence of adverse reactions, and patients are prone to relapse after withdrawal of the drug.^[13,14]^

Acupoint application, as a unique external treatment of traditional Chinese medicine (TCM), has been recommended for the treatment of AR since 2009.^[15]^ Acupoint application applies TCM into acupoints, such as pill, powder, ointment and so on, to prevent and treat diseases by using the dual effects of drug transdermal absorption and meridian effect.^[16]^ It has been used widely in China due to its non-invasive and easy to operate characteristics.^[17,18]^ Moreover, acupoint application has less adverse reactions and better efficacy compared with western medicine.^[19]^

Relevant research suggested that acupoint application could reduce allergic inflammation by inhibiting the expression of nerve growth factor (NGF) and its downstream pathways for AR patients.^[20]^ However, there is no systematic review and meta-analysis regarding the efficacy and safety of acupoint application combined with western medicine for AR. Thus, this study will assess the efficacy and safety of acupoint application combined with western medicine for AR.

## 2 Methods

### 2.1 Study registration

This protocol of systematic review and meta-analysis has been drafted under the guidance of the preferred reporting items for systematic reviews and meta-analyses protocols. Moreover, it has been registered on open science framework on July 3, 2020. (Registration number: DOI 10.17605/OSF.IO/NSGJH)

### 2.2 Ethics

Ethical approval is not required because there is no patient recruitment and personal information collection, and the data included in our study are derived from published literature.

### 2.3 Inclusion criteria for study selection

#### 2.3.1 Type of studies

Randomized controlled trials including acupoint application combined with western medicine for the treatment of AR will be included. The language will be limited to Chinese and English.

#### 2.3.2 Type of participants

All the included cases conform to the “Guidelines for diagnosis and Treatment of Allergic rhinitis,”^[21]^ regardless of nationality, race, age, gender, and source of cases.

#### 2.3.3 Type of interventions

The control group was treated with western medicine only, and western medicine type was not limited; the treatment group was treated with western medicine combined with acupoint application. The duration of treatment in both groups was not limited.

#### 2.3.4 Type of outcome measures

The main outcome measure was clinical efficacy. Efficacy evaluation criteria^[22]^ were calculated according to the formula (pre-treatment score - post-treatment score)/pre-treatment score ∗100%. Valid if more than 21%, invalid if ≤20. Efficient = effective/total number of cases ∗100%.

Secondary outcome measures were symptom score, serum IgE level, quality of life score of rhinoconjunctivitis quality of life questionnaire scale, and occurrence of adverse reactions.

### 2.4 Exclusion criteria

(1) The treatment group used other TCM therapies, such as acupuncture and moxibustion, TCM, etc;
(2) The outcome indicators of the original study did not meet the requirements;
(3) As for duplicate published literature, select the literature with the most complete data;
(4) Literature with incorrect or incomplete research data that cannot be obtained after contacting the author.

### 2.5 Search strategy

PubMed, EMbase, Cochrane Library, Web of Science, China National Knowledge Infrastructure, WanFang, the Chongqing VIP Chinese Science and Technology Periodical Database, and China biomedical literature database were searched by computer to collect randomized controlled trials of western medicine combined with acupoint application in the treatment of allergic rhinitis, and the retrieval time was from the establishment of each database to July 2020. At the same time, search Baidu, Google Scholar, International Clinical Trials Registry Platform, and Chinese Clinical Trials Registry to get more comprehensive data. Keywords were: “acupoint application,” “allergic rhinitis,” “allergic rhinitides,” et al. PubMed retrieval strategies are shown in Table 1.

**Table 1:**
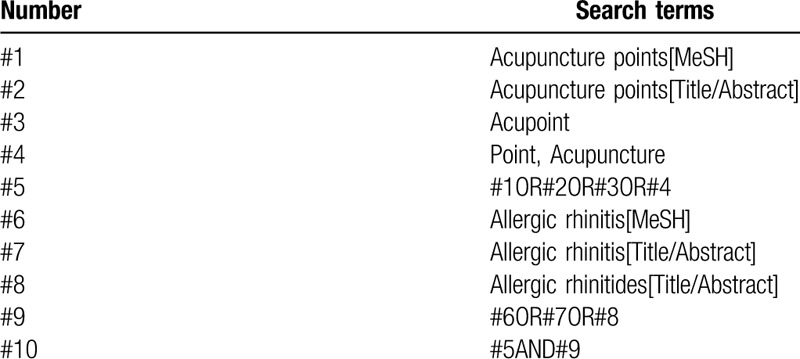
Search strategy in PubMed database.

### 2.6 Data extraction

**Figure 1:**
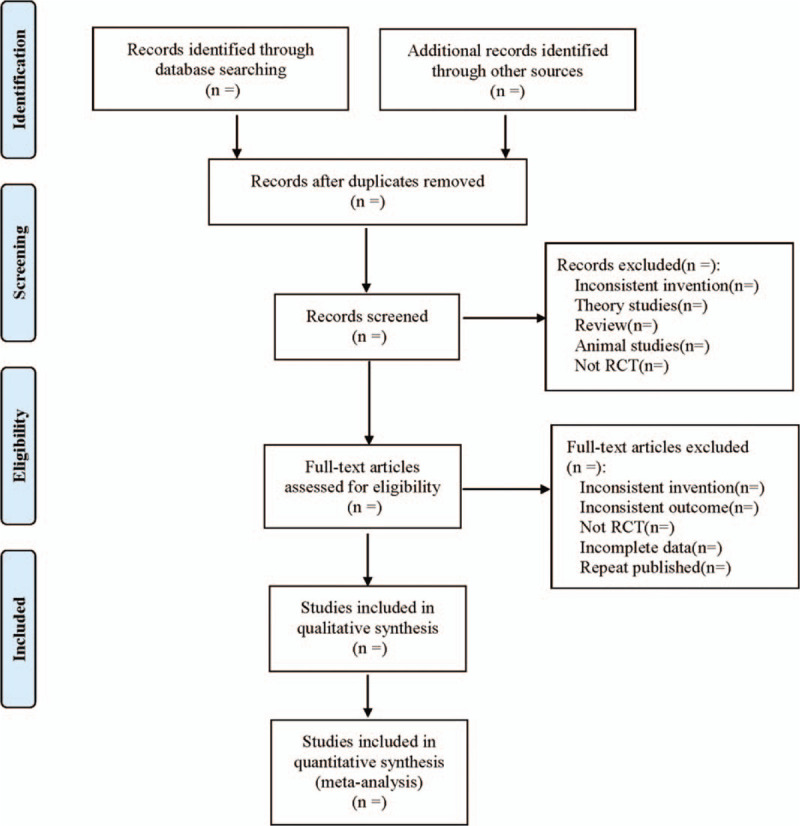
Flow diagram.

Endnote X7 was used for literature management. Two researchers independently screened the included literature according to the inclusion and exclusion criteria, and recorded the reasons for the exclusion. For example, in case of disagreement during the screening process, the author discussed with the third researcher. Excel 2019 was used to set up a data extraction table to extract data. The extraction contents were as follows: ① Included basic research information (study title, first author, publication time, sample size, sex ratio, average age, average course of disease, etc); ② Information about intervention measures (western medicine used in the treatment group and the control group, its dose, course of treatment, and acupoint applied in the treatment group, time of each acupoint applied, course of treatment, etc); ③ Risk evaluation items of bias in randomized controlled trials; ④ Related outcome indicators. The literature screening process is shown in Figure 1.

### 2.7 Risk of bias assessment

Two researchers independently evaluated the risk of bias in randomized controlled trials in accordance with the Cochrane Handbook of Systematic Reviewers, including the following items: random sequence generation, allocation concealment, blinding of participants and personnel, blinding of outcome assessment, incomplete outcome data, selective reporting, and other bias. The quality of studies was classified as being at of high, unclear or low risk of bias. In case of disagreement, a third researcher decided.

### 2.8 Statistical analysis

#### 2.8.1 Data synthesis

The RevMan 5.3 software provided by the Cochrane Collaboration was used for statistical analysis. ① Combined effect amount: Relative risk was selected as the statistic for the dichotomous variable. For continuous variables, Weighted Mean Difference was selected when the tools and units of measurement indicators are the same, Standardized Mean Difference was selected with different tools or units of measurement, and all the above were represented by effect value and 95% Confidence interval (CI). ② Heterogeneity test: Q test was used to qualitatively determine inter-study heterogeneity. If *P* ≥ 0.1, there was no inter-study heterogeneity, If *P* < 0.1, it indicated inter-study heterogeneity. At the same time, I^2^ value was used to quantitatively evaluate the inter-study heterogeneity. If I^2^ ≤ 50%, the heterogeneity was considered to be good, and the fixed-effect model was adopted. If I^2^ > 50%, it was considered to have significant heterogeneity, the source of heterogeneity would be explored through subgroup analysis or sensitivity analysis. If there was no obvious clinical or methodological heterogeneity, it would be considered as statistical heterogeneity, and the random-effect model would be used for analysis. Descriptive analysis was used if there was significant clinical heterogeneity between the 2 groups and subgroup analysis was not available.

#### 2.8.2 Dealing with missing data

If data is missing or incomplete, we will contact the corresponding author to obtain the missing data. If not, this study will be removed.

#### 2.8.3 Heterogeneity and subgroup analysis

In order to reduce the clinical heterogeneity between studies, subgroup analysis was conducted according to the age of the patients, which were divided into minors, adults and the elderly. Subgroup analysis was carried out for the included studies according to the types of western medicine, the time of acupoint application, and the course of medication.

#### 2.8.4 Sensitivity analysis

In order to test the stability of meta analysis results of indicators, a one-by-one elimination method will be adopted for sensitivity analysis.

#### 2.8.5 Reporting bias

For the major outcome indicators, if the included study was ≥10, funnel plot was used to qualitatively detect publication bias. Egger and Begg test are used to quantitatively assess potential publication bias.

#### 2.8.6 Evidence quality evaluation

The Grading of Recommendations Assessment, Development, and Evaluation ^[23]^ will be used to assess the quality of evidence. It contains 5 domains (bias risk, consistency, directness, precision, and publication bias). And the quality of evidence will be rated as high, moderate, low, and very low.

## 3 Discussion

TCM believes that allergic rhinitis belongs to the category of “Biqiu,” and is mostly the syndrome of deficiency in origin and excess in superficiality, and its incidence is mostly related to abnormal physical endowment, exogenous wind-cold, diet and fatigue, which lead to loss of lung function and weakness of spleen and kidney.^[24]^ TCM follows the concept of “treating cold syndrome with warming methods” and uses tonic and thermal drugs for treatment. Acupoint application therapy uses the different pharmacological effects of TCM to stimulate patients’ acupoints, so as to warm Yang and invigorate Qi, strengthening vital qi to eliminate pathogenic factors, dredge meridians, and dispel wind and cold.^[25]^ The medicines commonly used in acupoint application are: Bai Jie Zi (Semen Sinapis Albae), Xi Xin (Herba Asari), Sheng Jiang (Rhizoma Zingiberis Recens), Yuan Hu (Rhizoma Corydalis), etc, which have the function of warming the meridians and dispersing cold. The points commonly used for acupoint application are DU14 (Dazhui), BL12 (Fengmen), BL20 (Pishu) and BL13 (Feishu). DU14 (Dazhui) and BL12 (Fengmen) can dispel wind and cold. BL20 (Pishu) and BL13(Feishu) can invigorate the spleen to resolve dampness, ventilating lung qi for lowering adverse qi. Acupoint application can dispel the pulmonary wind and cold, and also regulate the function of lung, spleen and kidney.^[26]^ It has been shown ^[27]^ that acupoint application can regulate the TLR-NF-κB pathway and effectively reduce the sensitivity of TLR4 to foreign antigens, thus down-regulating the expression of NF-κB and reducing the release of inflammatory cytokines to achieve anti-inflammatory effects. According to the study,^[28]^ the clinical effective rate was 80.9% in 210 cases of allergic rhinitis treated by acupoint application, which can effectively improve the clinical symptoms of allergic rhinitis with small adverse reactions.

However, there is no systematic review and meta-analysis assessing acupoint application combined with western medicine for treating allergic rhinitis. This is the first protocol for systematic review and meta-analysis evaluating the efficacy and safety of acupoint application combined with western medicine for allergic rhinitis. This systematic evaluation and meta-analysis can provide evidence-based evidence for clinicians to use acupoint application in the treatment of allergic rhinitis. However, the study has some limitations. Due to different types of acupoints and different times of application, the results were affected and the bias was caused. In addition, we only search for articles in Chinese and English, which may cause certain publication bias.

## Author Contributions

**Data collection:** Yao Huang and Yihua Fan.

**Funding support:** Qinxiu Zhang.

**Literature retrieval:** Shasha Yang and Yue Ji.

**Software operating:** Chunying Tian and Mengni Zhang

**Supervision:** Chunying Tian and Qinxiu Zhang.

**Writing – original draft:** Yao Huang and Yihua Fan.

**Writing – review & editing:** Yao Huang and Qinxiu Zhang.
